# Brucellosis in ruminants and pastoralists in Borena, Southern Ethiopia

**DOI:** 10.1371/journal.pntd.0008461

**Published:** 2020-07-24

**Authors:** Bedaso Mammo Edao, Gobena Ameni, Zerihun Assefa, Stefan Berg, Adrian M. Whatmore, James L. N. Wood

**Affiliations:** 1 Disease Dynamics Unit, Department of Veterinary Medicine, University of Cambridge Madingley Road, United Kingdom; 2 Addis Ababa University, College of Veterinary Medicine, Bishoftu, Ethiopia; 3 Addis Ababa University, Aklilu Lemma Institute of Pathobiology, Addis Ababa, Ethiopia; 4 Department of Veterinary Medicine, College of Food and Agriculture, United Arab Emirates University, Al Ain, United Arab Emirates; 5 Animal and Plant Health Agency, Woodham Lane, Addlestone, United Kingdom; Faculty of Science, Ain Shams University (ASU), EGYPT

## Abstract

Brucellosis is a bacterial zoonotic disease that has important veterinary and public health consequences as well as economic impact in sub Saharan Africa including Ethiopia. A cross-sectional study was conducted in four selected districts of Borena Pastoral setting in Southern Ethiopia from October 2017 to February 2018 to estimate the prevalence of brucellosis and assess associated risk factors in cattle, sheep, goats and occupationally associated humans. A total of 750 cattle, 882 sheep and goats and 341 human subjects were screened for evidence of brucellosis using the Rose Bengal Test (RBT) with positive results confirmed by Competitive-ELISA(c-ELISA). Structured questionnaires were used for collection of metadata from individual animals, herders and animal attendants to test the association between explanatory and outcome variables. The overall animal level prevalence was 2.4% (95% confidence interval, CI: 1.4–3.7) in cattle, 3.2% (95% CI: 2.1–4.6) in sheep and goats, and 2.6% (95% CI: 1.2–5) in humans occupationally linked to livestock production systems. Herd size, parity, and history of abortion were risk factors associated with *Brucella* seropositivity (P<0.05) in cattle whereas in sheep and goats the results showed that district, age group, flock size, and history of abortion were significantly associated risk factors with *Brucella* seropositivity (P<0.05). Assisting calving and presence of seropositive animals in a household (P<0.05) were significantly associated with *Brucella* seropositivity in humans. Evidence of brucellosis in various animal species and the associated human population illustrates the need for a coordinated One Health approach to controlling brucellosis so as to improve public health and livestock productivity.

## Author summary

Brucellosis is a bacterial infectious disease with public health and economic importance mainly in low and middle-income countries (LMICs). The burden of this disease in livestock and its zoonotic importance in humans in sub-Saharan Africa including Ethiopia is poorly understood. In Ethiopia, although epidemiological studies were conducted in intensive dairy herds in high lands areas, there is shortage of data on the epidemiology and public health and impacts of brucellosis. This study was conducted to estimate the prevalence brucellosis in different species of livestock and pastoralists and thereafter to identify the potential risk factors affecting its occurrence and transmission. To this effect, a one health approach was used.

## Introduction

Brucellosisis is an economically important zoonotic disease of domesticated animals and humans, that can also affect wildlife. The disease is caused by Gram-negative bacteria of the genus *Brucella*. Of the six classical species of *Brucella* recognized, four are considered pathogenic to man. *Brucella melitensis*, which predominantly affects goats and sheep, is the most common cause of human brucellosis, whereas *B*. *аbortus*, found mainly in cattle, buffalo, elk, yaks, and camels, is the second most common cause of human infection. *B*. *suis*, which infects domestic pigs and rodents, and *B*. *canis* in canines are increasing in importance as sources of human brucellosis [1,2].

In resource-limited settings, including Ethiopia, disease control strategies are usually directed towards diseases with more dramatic impacts; programs featuring aspects of brucellosis intervention have generally not been launched. Consequently, brucellosis remains endemic and neglected, continuing to be a major public and animal health problem in developing regions of the world [3]. The disease can cause significant loss of productivity through abortion, prolonged calving, kidding, or lambing interval, low herd fertility, and comparatively low milk production in farm animals [4], and can cause chronic and febrile illness in humans.

In pastoral society brucellosis constitutes significant public health importance where close intimacy with animals, raw milk consumption and low awareness of zoonoses facilitate its transmission between livestock and humans. Milk is consumed raw by almost all pastoral communities, which is a threat for the pastoralists as it is the main source of infection with brucellosis [5].

Serological evidence of brucellosis in Borena pastoral region, Southern Ethiopia was reported by a few studies [6,7] These studies, however, had limited geographic coverage and none of them included parallel study on human brucellosis in the study area. Large numbers of human cases of brucellosis with fever, neurological complications and other generalized complications in rural and pastoral communities may be misdiagnosed and treated empirically as malaria or fever of unknown origin [8].

Cattle, camels, goats, and to some extent sheep are the principal livestock species that are reared by Borena pastoralists. Herding of these animals together, which is the normal practice of the traditional pastoral people, is one of the putative factors of transmission of *Brucella* infection. Comprehensive studies on brucellosis in different animal species sharing the same ecological zone, and zoonotic significance in occupationally linked humans are scarce. Therefore, documenting the risk profile of human–animal interface in Borena pastoral setting is vital in developing feasible control strategies in Ethiopia. Hence, the objectives of this study were to determine the prevalence of brucellosis in cattle, sheep and goats, and their attendants using serological tests (RBT and c-ELISA), identify potential risk factors precipitating the disease and assess the knowledge, attitude and practices (K-A-P) of herders and animal attendants so as to assess public health significance.

## Materials and methods

### Study setting

**Fig 1:**
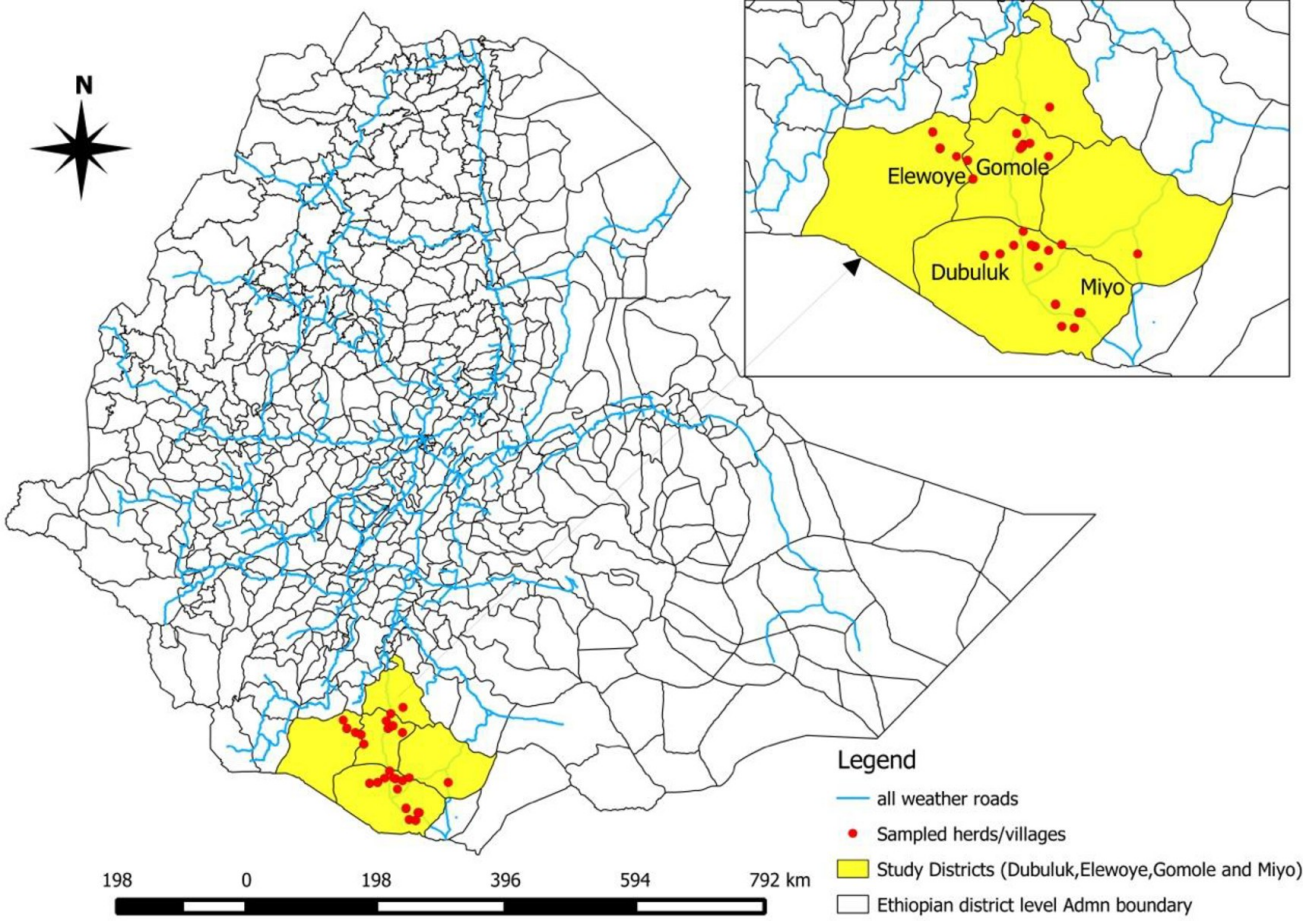
Map of Ethiopia and Borena pastoral zone. This map is our own developed from Ethiopian shape files using QGIS Software, 2013. The yellow shaded region represents study districts and red dots represent sampled villages.

Borena pastoral area is located in Oromia Regional state, Southern Ethiopia. The capital of the zone, Yabello, is 575 km south of Addis Ababa. According to the Borena Zone Pastoral Development Office [9], the zone has recorded livestock populations of 1,416,180 cattle, 1,262,782 goats, 776,870 sheep, 237,205 camels, 306,057 poultry, 102,767 donkeys, 1,841 horses and 4,433 mules; the human population was 1,283,925 in 2015 [10]. Borena Zone comprises thirteen districts and borders Kenya in the southern part at Moyale, Miyo, Dirre and Teltelle districts. The study was conducted in four randomly selected districts; Gomole, Elewoye, Dubuluk, and Miyo. A map of our study area is shown in Fig 1.

Generally, the Borena plateau represents a lowland area where altitude gently slopes from the North (1650 m) to the South (1000 m) above sea level. The area has a bimodal rain pattern with annual average precipitation ranging from 300 mm to 700 mm. The main rainy season (65% of precipitation) extends from March to May, and a minor rainy season is between mid-September and mid-November. The main dry season extends from December to February [11]. As surface water is very scarce in the area, deep wells, shallow ponds, and large machine-excavated ponds are important sources of water for both livestock and humans. Clans own traditional wells, while large ponds are communal and often responsible for aggregation of large numbers of animals at the water points.

The livestock production system is predominantly extensive, where animals are allowed to forage freely during daytime and kept in open enclosures during the night. [12]. Livestock share common grazing areas and watering points, and probably mingle at villages although separate enclosures are used for each species. Mobile herds are often maintained together with five or more village herds to reduce labour demand, a condition that facilitates transmission of the disease from infected to susceptible herds [6].

The pastoral village, *Olla* in Borena, is characterized by the clustering of households with close proximity of houses in a pastoral camp. A village chief, Abba Olla, who is an important contact person in facilitating cooperation between livestock owners, traditionally administers each village, which usually varies in size between 7 and 20 households. Keeping multiple livestock species and seasonal herd mobility are part of the dynamic nature of the pastoral production system. Livestock constitute the principal source of livelihood for Borena households. Nearly 70% of household cash revenues come from pastoral sources, mainly from livestock sales with sales from dairy products constituting only a small proportion [11].

### Study design

A cross-sectional study was conducted to determine the prevalence of *Brucella* infection in cattle and sheep and goats and occupationally associated animal owners and attendants in four districts of the Borena Pastoral region, identify the potential risk factors associated with the seropositivity and to assess the KAP of visited household members towards brucellosis. After collecting the list of number of districts in Borena pastoral zone, the four districts (Gomole, Elewoye, Dubuluk, and Miyo) were selected randomly based on livestock population size and species diversity, and close geographic location to a regional veterinary laboratory. Study animals were grouped into different categories based on their sex, age, herd or flock size, physiological status and presence or absence of reproductive problems such as abortion history. Age determination and history for presence or absence of reproductive problems were obtained from animal owners and attendants. The target pastoral associations (PAs) or villages from the four districts were selected based on presence of at least three livestock species, accessibility of villages by vehicle and proximity of the villages to the main roads. Cattle and sheep and goats above six months of age were recruited for this study. Relevant individual animal biodata and herd level information were collected using a semi-structured questionnaire. Demographic information of voluntary participants and their KAP related to brucellosis were also recorded using a pretested structured questionnaire.

### Study populations

#### Cattle, sheep and goats

The target populations of cattle and sheep and goats were composed of local cattle breeds of Boran type, blackhead Somali sheep, and the long-eared Somali goats. Putative biological factors believed to be associated with epidemiology of brucellosis were recorded. These included, sex, age, species, herd size and physiological status.

#### Humans

Household members who had frequent close contact with animals and animal products for at least one year from the selected pastoral associations (PAs) or villages in the study area were sampled. A trained medical laboratory technologist from Yabello hospital was used for this purpose. After the purpose of the study was explained and consent to participate in the study was obtained from participants, blood samples were collected from volunteer livestock owners and animal attendants.

### Sample size determination

A multistage sampling combined with the convenient sampling strategy was employed for sampling of individual animal species. A PA or a village is the smallest administrative unit in the study district. The PAs for the study were selected by randomization after obtaining the total number of PAs in the district. The total number of PAs within the four selected districts in Borena zone were listed and used as a sampling frame. Households with two or more livestock species were identified and approached for permission to sample their animals. Factors such as presence of three animal species per village, species of animals per household, willingness of herders to cooperate, and availability of herds during the visit were taken into consideration to estimate the number of each animal species to be sampled per village. Thus sample size (n) was determined based on the formula previously published [13].

The average livestock holding per household was estimated to be 20 cattle, 15 goats, 6 sheep and 10 camels with possible variation between ethnic groups [11]. As a result, with expected prevalence of 10.6% in cattle [6], and 9.7% in sheep and goats [14] with 5% desired absolute precision at 95% confidence level was assumed to calculate the desired samples size in cattle and sheep and goats. Accordingly, a minimum sample size of 150 cattle, 134 sheep and goats were required to be sampled from each of the four districts. Hence, this minimum target was reached by serum sampling a total of 750 cattle and 882 sheep and goat from the targeted villages. Similarly, with the expected prevalence of 3.7% in humans [15] with 5% desired absolute precision at 95% confidence level, a total of 341 blood samples were collected from occupationally linked humans.

### Sample collection and laboratory tests

Blood samples (10ml) were collected from cattle, sheep and goats from the jugular vein and transported to Yabello Regional Veterinary Laboratory and stored at 4°C. The following day the blood samples were centrifuged at 1500 × g for 10 min to obtain the serum. Sera were decanted into cryovials, identified and stored at −20°C until being transported in cold chain using ice packs.

#### Rose Bengal test (RBT)

All sera samples collected were initially screened by RBT using RBT antigen (Animal and Plant Health Agency (APHA), United Kingdom) according to described procedures [16]. Briefly, sera and antigen were taken from refrigerator and left at room temperature for half an hour before the test to reach room temperature. RBT antigen (30 ml) was added onto a clean plate next to an equal volume of test serum sample (cattle and human). For sheep and goats, in order to improve the sensitivity of RBT as previously recommended [17], one volume of antigen and three volumes of serum (e.g. 25ul with 75ul) was used instead of an equal volume of each. The antigen and test serum were mixed thoroughly with a plastic applicator, shaken for 4 min, and the result (presence of agglutination or not) was read immediately.

#### Competitive ELISA

All RBT positive sera were further tested at Addis Ababa University, Aklilu Lemma Institute of Pathobiology (AAU-ALIPB) using the COMPELISA 160 and 400, a competitive ELISA kit for the detection of antibodies against *Brucella* in serum samples (Animal and Plant Health Agency, Addlestone, United Kingdom). The test was performed according to the manufacturer’s instructions.

### Case definition

An animal or human case was considered positive if it tested seropositive on both RBT and c-ELISA in serial interpretation. Similarly, a herd or flock was considered seropositive when at least one animal in a herd or flock tested positive. Since there is no history of vaccination against brucellosis in Ethiopia, seropositivity observed in this study was considered to be due to natural infection of *Brucella*.

### Ethics approval and consent to participate

Ethical clearance for this study was obtained from Aklilu Lema Institute of Pathobiology, Addis Ababa University, Minutes of Institutional Research Ethics and Review committee (Minute number: ALIPB/IRB/011/2015/16. This committee followed the protocols of the National Research Ethics Review Guideline formulated by the Ministry of Science and Technology of the Federal Democratic Republic of Ethiopia in 2014. Before conducting the research, participants were informed of the objectives of the study and written, and signed consent was obtained from the livestock owners and occupationally linked household members to collect blood samples for testing them for antibodies against *Brucella* infection. When participants were illiterate, informed verbal consent was obtained. For participants younger than 18 years, consent was obtained from their guardian.

### Data analysis

Data generated from the survey and laboratory investigations were recorded and coded using a Microsoft Excel spread sheet (Microsoft Corporation) and analyzed using STATA version 15.0 for Windows (Stata Corp. College Station, TX, USA). The association between explanatory and outcome variables was analyzed at individual animal level by using univariable and multivariable logistic regression. A multivariable logistic regression model was used to identify risk factors associated with *Brucella* infection, at individual and herd or flock level, keeping village as the cluster variable. Variables with a p-value less than or equal to 0.05 (in univariable analysis) were included in the multivariable logistic model. For variables that showed strong co-linearity (p<0.05), one of the two variables was excluded based on biological plausibility to *Brucella* infection. Further selection of variables in the final model was based on stepwise backward elimination procedure. Prevalence in cattle, sheep and goats, as well as in humans, was compared with the chi-square and Fisher’s exact test as appropriate. Odds ratio was used to assess the strength of association between exposure variables associated with seropositivity of the disease in both animals and human.

## Results

### Descriptive statistics of sero-prevalence

The overall animal seroprevalence of brucellosis was 2.8% (CI = 2.1–3.7) in ruminants. When species of ruminants was considered, animal seroprevalence was 2.4% (CI = 1.4–3.8) in cattle, and 3.2% (CI = 2.1–4.6) in sheep and goats (Table 1). Furthermore, the seroprevalence of brucellosis was 2.6% (95% CI = 1.2–4.9) in occupationally exposed individuals.

**Table 1:** Univariate logistic regression analysis of the risk factors for *Brucella* seropositivity in cattle.

Risk factor	Level	N^o^ Sampled	N^o^ Positive (%)	OR	95% CI	p-value
District	Dubuluk	191	3 (1.6)	-	-	
	Eleweye	160	11(6.8)	4.6	1.3–16.8	0.02
	Gomole	136	4(2.9)	2.0	0.4–8.6	0.4
Herd Size	<20	165	4(2.4)	-	-	
	20–50	267	8(3.0)	1.2	0.4–4.2	0.72
	>50	55	6(10.9)	4.9	1.3–18.2	0.01
Age	≤ 5	171	2(1.2)	-	-	
	> 5	316	16(5.1)	4.5	1.0–19.8	0.04
Physiology	Heifer	68	2(2.9)	-	-	
	Lactating	242	8(3.3)	1.1	0.2–5.4	0.88
	Not pregnant	119	3(2.5)	1.0	0.1–5.2	0.86
	Pregnant	57	5(8.7)	3.2	0.6–17.0	0.17
Parity	≤ 2	285	5(1.8)	-	-	
	> 2	202	13(6.4)	3.8	1.4–11.0	0.01
Abortion	No	407	11(2.7)			
	Yes	80	7(8.8)	3.5	1.3–9.1	0.01

The highest (3.7%) animal seroprevalence was recorded in goats, followed by cattle (2.4%) and sheep (1.4%). There was variation in the distribution of seroreactor animals and humans among the four districts. Eleweye district had the highest proportion of seropositive in cattle (6.3%) as well as in sheep and goats (6.1%). Furthermore, the same District had the highest (5.1%) seroprevalence in humans. On the other hand, the seroprevalence was nil in cattle in Miyo district while seroprevalences of 4% and 2.3% were recorded in sheep and goats and humans in Miyo district, respectively (Table attached as supplementary document **S1 Table**).

When pastoral villages are considered, seropositive animals were found in 60% (12/20) and 15% (3/20) of the villages with at least one and two positive animal species, respectively. Village level seropositive reactors were more frequently detected in sheep and goats (23.3%) than in cattle (11.4%). The average number of positive animals per positive herd was generally low, 1.4 in both cattle, sheep and goats, suggesting a slow within herd transmission of the disease. The seroprevalence ranged from 0–23% in sheep and goats and 0–11.4% in cattle. In sheep and goats, the highest seroprevalence was recorded in Saba, 23.3% followed by Rarewardelle, 12%. The highest seroprevalence in cattle was also recorded in Saba, 11.4% followed by 6.7% in Harobake. On the other hand, the seroprevalence in humans across the pastoral villages ranged from 0–22.2% with the highest also being in Saba village (Table attached as supplementary document **S1 Table**).

### Risk factors for *Brucella* spp. seropositivity in cattle

Table 1 shows the prevalence and univariate logistic regression analysis of associations of risk factors for *Brucella* seropositivity in cattle. The number of animals from Miyo district was excluded from the final model as all tested animals from this district were seronegative for *Brucella* infection. The major exposure variables that were considered to predict the response of the outcome variable includes, district, herd size, age, parity, physiological status, and history of abortion. The result showed that most of the recorded variables showed a high degree of association with seropositivity to *Brucella* infection.

The variables with a p-value <0.05 from univariable logistic regression analyses were included in the final multivariable logistic model. Two variables, district and age of animals that showed co-linearity with other explanatory variables (district with herd size and age with parity) were not included in the multivariable logistic regression model.

The final multivariable logistic regression model (Table 2) showed that animals kept in a large herd were more likely to be exposed to *Brucella* infection than those maintained in a medium and small herd (OR = 6.3, 95% CI = 1.6–24.8, P = 0.01). The result also showed that animals with parity greater than two were more likely to acquire infection than those with parity less than two (OR = 4.8, 95% CI = 1.6–14.5, P = 0.004). Similarly, cows with history of abortion were more likely to be seropositive for *Brucella* infection than cows without such history (OR = 3.7, 95% CI = 1.3–10.4, P = 0.000).

**Table 2:** Multivariable logistic regression model of risk factors for *Brucella* seropositivity in cattle at individual and herd level using village as a cluster variable.

Risk factor	Level	N^o^ sampled	N^o^ positive (%)	OR	95% CI	p-value
Herd Size	< 20	165	4(2.4)	Ref		
	20–50	267	8(3.0)	1.3	0.3–4.5	0.67
	> 50	55	6(10.9)	6.3	1.6–24.8	0.01
Parity	≤ 2	285	5(1.8)	Ref		
	> 2	202	13(6.4)	4.8	1.6–14.5	0.004
Abortion	No	407	11(2.7)	Ref		
	Yes	80	7(8.8)	3.7	1.3–10.4	0.01

### Risk factors for *Brucella* spp. seropositivity in sheep and goats

The prevalence and univariate logistic regression analysis of associations of explanatory variables for *Brucella* seropositivity in sheep and goats was shown in Table 3. Seropositivity was found to be significantly associated with district, age > 3 years, increased flock size, and with history of abortion (P< 0.05)

**Table 3:** Univariate logistic regression analysis of the risk factors for *Brucella* infection in sheep and goats.

Risk factor	Level	N^o^ Sampled	N^o^ Positive (%)	OR	95% CI	p-value
District	Dubuluk	219	3 (1.4)	Ref	-	
	Eleweye	213	13 (6.1)	4.7	1.3–16.7	0.01
	Gomole	155	2 (1.3)	1.0	0.2–5.7	0.9
	Miyo	230	10 (4.4)	3.4	0.8–12.1	0.06
Flock Size*	< 39	411	7 (1.7)	Ref	-	
	≥ 39	406	21 (5.1)	3.1	1.3–7.4	0.01
Species	Ovine	196	3 (1.5)	Ref		
	Caprine	621	25 (4.0)	2.7	0.8–9.0	0.11
Age	≤ 3 Years	243	2 (0.8)	Ref	-	
	> 3 Years	574	26 (4.5)	5.7	1.3–24.3	0.01
Physiology	Weaner	51	1 (2.0)	Ref	-	
	Lactating	449	9 (2.0)	1.0	0.1–8.2	0.98
	Not Pregnant	89	3 (3.4)	1.7	0.2–17.2	0.63
	Pregnant	228	15 (6.6)	3.5	0.5–27.3	0.23
Parity	≤ 2	221	4 (1.8)	Ref	-	
	> 2	596	24 (4.0)	2.3	0.8–6.6	0.13
Abortion	No	538	10 (2.0)	Ref		
	Yes	279	18 (6.5)	3.6	1.6–8.0	0.001

* Median flock size was 39.

Explanatory variables with P<0.05 in univariate logistic regression analyses were subjected to a multivariate logistic regression model. Variables such as district, flock size, age, and history of abortion were included in the multivariable logistic regression model (Table 4). Thus, further selection of variables in the final model was based on stepwise backward elimination procedure. The multivariable logistic regression model indicated that sheep and goats from Eleweye district were 6 times more likely to be seropositive for *Brucella* infection than other districts of the study area (OR = 6.0, 95% CI = 1.7–22, P = 0.006). Increase in flock size ≥ 39 was significantly associated with *Brucella* seropositivity (OR: 3.3, 95% CI = 1.3–8.4, P = 0.01). Mature animals (> 3 years) were 4.8 times more likely to be seropositive for *Brucella* infection than young sheep and goats (OR = 4.8, 95% CI = 1.1–20.7, P = 0.04). Having a history of abortion was significantly associated with *Brucella* seropositivity (OR = 3.1, 95% CI = 1.4–6.9, P = 0.006).

**Table 4:** Multivariable logistic regression analysis of the risk factors for *Brucella* infection in sheep and goats.

Risk factor	Level	N^o^ Sampled	N^o^ Positive (%)	OR	95% CI	p-value
District	Dubuluk	250	3 (1.2)	-	-	
	Eleweye	215	13 (6.1)	6.0	1.7–22.0	0.006
	Gomole	166	2 (1.2)	1.6	0.3–10.2	0.6
	Miyo	251	10 (4.0)	3.0	0.8–11.3	0.11
Flock Size	< 39	432	7 (1.6)	-	-	
	≥ 39	450	21 (4.7)	3.3	1.3–8.4	0.01
Age	≤ 3 Years	292	2 (0.7)	-	-	
	> 3 Years	590	26 (4.4)	4.8	1.1–20.7	0.03
Abortion	No	538	10 (2.0)	-		
	Yes	279	18 (6.5)	3.1	1.4–6.9	0.006

### Serological survey for human Brucellosis

Seroprevalence of brucellosis in occupationally linked household members and its association with demographic factors in the four districts using Fishers exact test is shown in Table 5. An individual seroprevalence of 1.5% (n = 5) in Eleweye, 0.6% (n = 2) in Miyo, and 0.3% (n = 1) in both Dubuluk and Gomole districts were recorded. Relatively higher seroprevalence was observed in male individuals, 1.5% (n = 5) versus 1.2% (n = 4) in females, and in age group 20–60 years compared to other age groups. Married individuals had highest seropositivity, 2.4% and majority (77.7%) of participants were illiterate and had the highest seropositivity of 2.4%. Households with 1–5 people and with more than three animal species had highest seroprevalence at 1.5% and 2.6%, respectively.

**Table 5:** Univariable logistic regression analysis of K-A-P related risk factors for *Brucella* seropositivity in humans.

Risk factor	Level	N^o^ Sampled	N^o^ Positive	OR	95% CI	p-Value
District	Dubuluk	93	1(0.3)	-	-	
	Eleweye	98	5 (1.5)	3.6	0.6–22.6	0.17
	Gomole	61	1 (0.3)	1.5	0.2–15.0	0.72
	Miyo	89	2 (0.6)	1.7	0.2–13.6	0.59
Consume raw milk	No	54	1 (1.8)	-	-	
	Yes	287	8 (2.8)	1.1	0.2–6.3	0.93
Consume raw meat	No	181	4 (2.2)	-	-	
	Yes	160	5 (3.1)	1.4	0.4–5.0	0.61
Consume raw milk mixed with blood						
	No	139	1 (0.7)	-	-	
	Yes	202	8 (4.0)	4.0	0.7–23.2	0.12
Assist during birthing/calving						
	No	198	1 (0.5)	-	-	
	Yes	143	8 (5.6)	8.3	1.4–47.5	0.02
Dispose dead foetus or RFM*						
	No	171	2 (1.2)			
	Yes	170	7 (4.1)	3.6	0.7–13.2	0.12
Seropositive animals at household						
	No	317	3 (1.0)			
	Yes	24	6 (25.0)	31.5	7.9–126	0.00

* Retained foetal membranes

On univariate logistic regression analysis, assisting during calving or birthing (P = 0.02) and presence of seropositive animal at household (P = 0.000) were significantly associated with increased risk of brucellosis in humans (Table 6). Participants from Eleweye districts were 3.6 times more likely to be seropositive for *Brucella* infection than other districts in the study area. Individuals who consumed raw milk mixed with blood had 4 times higher odds of *Brucella* seropositivity than those who had not (OR = 4.0, 95% CI = 0.7–23, although this was not significant). Similarly, household members who disposed of foetal material were 3.6 times more likely to be seropositive for *Brucella* infection (OR = 3.6, 95% CI = 0.7–13), but again this was not significant.

On multivariate logistic regression analysis, assisting during calving or birthing and presence of seropositive animal at household were significantly associated with increased risk of *Brucella* seropositivity in humans (p<0.05) (Table 6).

**Table 6:** Multivariate logistic regression analysis of factors associated with brucellosis in humans.

Risk factor	Level	N^o^ Sampled	N^o^ Positive (%)	OR	95% CI	p-value
Consume raw milk with blood						
	No	139	1 (0.7)	-	-	
	Yes	202	8 (4.0)	6.0	0.7–50.4	0.098
Assist during birthing/calving						
	No	198	1 (0.5)	-	-	
	Yes	143	8 (5.6)	9.9	1.4–72.0	0.024
Dispose dead foetus or RFM*						
	No	171	2 (1.2)			
	Yes	170	7 (4.1)	3.4	0.7–19.1	0.169
Seropositive animal at household						
	No	317	3 (1.0)			
	Yes	24	6 (25.0)	45.1	8.7–233.5	0.000

* Retained foetal membranes

## Discussion

The present study documented serological evidence of brucellosis in cattle, sheep and goats and occupationally exposed household members in four selected districts of Borena pastoral region in Southern Ethiopia. As no single serological test is appropriate in all epidemiological situations, the use of two tests applied serially is usually recommended for maximal specificity and ruling out of false positive cross-reactions [17,18]. A combination of RBT and c-ELISA or the Complement Fixation Test (CFT) is the most widely used serial testing scheme. In cattle and humans, we used a combination of RBT and C-ELISA, and for sheep and goats, a modified RBT and C-ELISA was used serially. RBT is selected as a screening test based on low cost, easy performance and high sensitivity, especially in endemic areas [19]. However, C-ELISA is selected due to its high specificities to discriminate between false positive cross–reactions and *Brucella* infections [20,21]. False positive serological reactions in RBT could be due to cross-reactions with smooth lipopolysaccharide (S-LPS) antigens of other Gram-negative bacteria. As there has never been history of vaccination in Ethiopia, seropositivity in all cases is due to natural infection.

The animal level prevalence of 2.4% detected in cattle in the present study was comparable with the report of 2.9% by Jergefa et al [22] in central Ethiopia, and 3.1% by Ibrahim et al [23] in Jimma zone, Southwest Ethiopia. However, a relatively lower prevalence of 1.4% by Gumi et al [24] from Guji and Somali pastoral region, 1% by Adugna et al [25] from mixed crop livestock production in Western Ethiopia, and 1.3% by Degefu et al [26] from Agro–pastoral region in Somali regional state, was reported. On the other hand, a consistent prevalence with the present study was reported in Ethiopia by Asgedom et al [27] who reported a prevalence of 2.4% in cattle in Alage district. Higher prevalence than the present study was reported in Western Tigray [28], Borena [6] and in other African countries [5,29,30]. The difference in the prevalences recorded in the different study area may be associated with the differences in agro ecology, management system, tests used to detect *Brucella* seropositivity and sample sizes used in each study.

Our finding of 16% herd level seroprevalence in cattle was similar to 15% reported by Ibrahim et al [23] and 13.6% by Jergefa et al [22] whilst other studies in Ethiopia showed a lower seroprevalence [25,31–33]. Conversely, other authors have reported higher herd level seroprevalences; 45.9% from Ethiopia by Kebede et al [34], 55.5% from Uganda by Bernard et al [35] and 62% from Zambia by Muma et al [19]. Such contrasting findings could be either related to the overall individual animal level prevalence status of the disease or the size of studied herds.

The overall individual animal level seroprevalence of *Brucella* infection in small ruminant recorded in this study was similar to previous studies by Teklue et al [36] and Tsehay et al [37] who reported prevalence of 3.5% and 3.6% in small ruminant in southern Tigray and Somali pastoral region, respectively. Conversely, in Afar pastoral region, a higher individual animal level prevalence of 12.4% and 13.7% were reported by Tegegn et al [38], and Tadeg et al [39], respectively. The flock level seroprevalence of 22.7% recorded in the present study was comparable to the findings of Deddefo et al [40] in Arsi and East Shoa zones, central Ethiopia and Asmare et al [41] in pastoral regions of Guji and Borena, Southern Ethiopia. The differences in seroprevalences observed could be due to variations in sensitivity and specificity imparted by the various test used, agro-ecological location, and sample size and production systems.

Larger herd or flock sizes were found to be significantly associated with *Brucella* seropositivity in cattle and small ruminants, as previously reported [23,25,36,41–43], and can be explained by the fact that an increase in herd size is usually accompanied by an increase in stocking density, one of the determinants for exposure to *Brucella* infection especially following abortion calving [44].

Association of *Brucella* seropositivity with increase in cattle parity number greater than two was consistent with the findings of earlier studies [29,32,45]. Similarly, adult sheep and goats (>3 years) were more likely to be seropositive than younger animals as previously reported [8,41,46–48], This has been attributed to increased chance of infection with increasing age [49]. Seroprevalence of brucellosis may increase with age as a result of prolonged duration of antibody responses in infected animals and continued exposure to pathogen, particularly in pastoral production systems where animals are maintained in herds over long period of time. In our data analysis, the fact that older animals showed higher seropositivity to *Brucella* infection than young ones, and this variable (age) showed collinearity with parity substantiates this fact.

Reproductive loss due to abortion, birth of weak offspring, and infertility are recorded as the common clinical signs of brucellosis in natural hosts [50,51]. The major complaints of abortion in farm animals is ascribed to *Brucella* infection [5,19,52]. In this study, seropositivity to *Brucella* infection in cattle, sheep and goats was significantly associated with history of abortion as previously reported in Ethiopia [23,33,53] and Uganda [54].

In general, the distribution of *Brucella* antibodies among different districts, animal species and pastoral villages was found to be variable. This could be associated with variability of the herd sizes and samples tested per visited households. Short drought cycles caused by climate changes drive Borena pastoralists to trek their livestock, with the exception of lactating and few pregnant animals, to different villages, districts, or even crossing national borders by traveling several kilometres. This results in massive concentration of animals in areas with relatively better pasture and watering points. This in turn, may contribute to the increased transmission of *Brucella* organisms among different herds resulting in emergence of new infectious foci creating variation in distribution of *Brucella* infections among different villages and districts. Mobility also increases the opportunity of interactions with wild animals. Sharing the same ecology with wildlife was shown to be an important risk factor for brucellosis in domestic animals kept under traditional livestock production systems [19,55].

Even though attempts to isolate *Brucella* species circulating in the region were not successful, these results reveal more than one seroreactor animal species in villages and household visited. While the possibility of the transmission of *Brucella* species outside the ‘preferred’ host cannot be ruled out [56–58] particularly when animal species mix so freely, this may suggest that both *B*. *abortus* and *B*. *melitensis* circulate in this pastoralist population as recently shown in neighbouring Tanzania [59].

In the present study, an overall human *Brucella* seropositivity of 2.6% (95% CI = 1.2–4.0) was recorded (Table 1). This finding is comparable with previous reports in Sidama zone, Southern Ethiopia [15] and in Adami Tullu Jido Kombolcha district, central Ethiopia [60]. Similar findings were also reported in Eritrea [61] and Chad [5]. Conversely, a higher seroprevalence than the current study was recorded in Kenya [62]. The variations observed in different studies could be associated with the degree of endemicity of brucellosis in the livestock population, duration of exposure, sample size epidemiological settings of the study population and variability related to diagnostic test and method applied.

The present study determined risk factors for human brucellosis among occupationally linked household members in Borena pastoral region. Studies in Kenya by Namanda et al [63] and in Tanzania by John et al [64] have reported occupation as a risk factor for acquiring brucellosis, whereby animal handlers and associated professionals were the most susceptible groups. In our study, it was revealed that 98.8% of participants had no knowledge of brucellosis. Therefore, there is a clear need to promote health education about transmission, prevention and risk factors for brucellosis to occupational risk groups to reduce the risk of acquiring the disease.

Consumption of unpasteurized milk was reported to be a risk factor for acquiring brucellosis in human [63,65,66]. Practices of consuming raw milk among Borena pastoral communities is due to a belief that boiling a milk would reduce its nutritional content. Our study indicated that 84% (n = 287) of participants had consumed raw milk, 59% (n = 202), raw milk mixed with blood collected from domestic livestock, and 47% (n = 160) consumed raw meat. However, none of these practices were significantly associated with seropositivity, although numbers were low. Variations in number of human seroreactors among the four districts followed the same pattern as seropositivity in cattle and goats, although again, results were not significant.

The multivariable logistic regression analysis of potential risk factors indicated that assisting during birthing or calving was significantly associated with *Brucella* seropositivity (OR = 9.9, 95% CI = 1.4–72). Assisting in calving or birthing was associated with increased risk of brucellosis in similar settings in Northern Tanzania [67] and in Kenya [68]. *Brucella* species are known to have a predilection for reproductive organs particularly placenta and aborted foetuses, it is reasonable that assisting animals in delivery would increase risk of infection [62].

Our study revealed that human seropositivity was associated with presence of seropositive animal at household. The odds of human seropositivity were 45 times higher in households with a seropositive animal as compared to those without. Similar finding were reported in Kenya by Osoro et al [62] and Kyrgyzstan by Bonfoh et al [69]. This study thus contributes to the evidence base that human brucellosis is often transmitted from livestock in close contact [29,70].

In many developing countries including Ethiopia, brucellosis continues to be a major public and animal health problem as there is no control strategies put in place, although a One-Health strategy is now being developed in Ethiopia.

### Limitation of the study

This study has some limitations. Seasonal migration of livestock in Borena in search of good pasture and watering points could be associated with temporal variation of prevalence of the disease that was not assessed due to the cross-sectional design of the current study. Children less than 5 years of age were not included in the study that may limit the representation of the data to the entire population. Security problems related to political instability limited number of districts surveyed. As the survey was conducted in drought season, some of pastoralists refused to allow their herds to be sampled contending that collecting blood sample from their animals could impede productivity.

### Conclusion

The current study revealed that antibodies to *Brucella* spp. are detected in cattle, sheep and goats sharing the same ecological zone and occupationally linked pastoralists in Borena, Ethiopia. The study also showed associations between human and animal seropositivity at household level. Adult age group, larger herd/flock sizes, greater parity in cattle and history of abortion were found to be risk factors for brucellosis in cattle and sheep and goats. Assisting during calving without using protective equipment was also an explanatory variable associated with *Brucella* seropositivity in humans. The traditional mixed livestock farming system in Borena supplemented with recurrent livestock mobility triggered by climatic changes and other factors will likely continue to enhance the endemicity of the disease in the area. The occupational risks for pastoralists such as contact with infected animals, particularly assisting during calving without protective equipment and the tradition of raw dairy product consumption facilitates zoonotic transmission. Further extensive epidemiological studies involving one health approach needs to be undertaken to isolate and characterize circulating *Brucella* species among humans and livestock so as to identify the transmission dynamics of *Brucella* species. Raising public awareness regarding traditional practices that could potentially cause exposure to *Brucella* infection and prevention methods is a clear need. A socioeconomic study to provide a societal perspective of the burden of the disease is highly warranted as this would help in determining feasible control measures to be undertaken in different settings.

## Supporting information

S1 Table Supplementary tables. (DOCX)

S1 Text Ethical Approval. Approval from Addis Ababa University, Aklilu Lemma Institute of Pathobiology, Institutional Review Board.
(PDF)

S1 Dataset Cattle raw data set used for analysis. (XLSX)

S2 Dataset Small ruminants (sheep and goats) raw data set used for analysis. (xlsx).
(XLSX)

S3 Dataset Human raw data set used for analysis. (XLSX)
