# Supplementary material for: Brucellosis in ruminants and pastoralists in Borena, Southern Ethiopia
Source: PLoS Negl Trop Dis. 2020 Jul 24;14(7):e0008461. doi: 10.1371/journal.pntd.0008461 (PMC7406081; doi:10.1371/journal.pntd.0008461)
Supplement: S1 Table — (DOCX) [file pntd.0008461.s001.docx]

**Table 1**: Distribution of seroreactor animals and humans among the four districts in Borena Zone, Southern Ethiopia (serial interpretation of RBT and C-ELISA).

| District | Species tested | N^o^ Sampled | N^o^ Positive (%) |
| --- | --- | --- | --- |
| Dubuluk | Cattle | 214 | 3 (1.4) |
|  | Sheep and goats | 250 | 3 (1.2) |
|  | Human | 93 | 1 (1.1) |
| Eleweye | Cattle | 176 | 11 (6.3) |
|  | Sheep and goats | 215 | 13 (6.1) |
|  | Human | 98 | 5 (5.1) |
| Gomole | Cattle | 140 | 4 (3.0) |
|  | Sheep and goats | 166 | 2 (1.2) |
|  | Human | 61 | 1 (1.6) |
| Miyo | Cattle | 220 | 0 (0) |
|  | Sheep and goats | 251 | 10 (4.0) |
|  | Human | 89 | 2 (2.3) |
| Total | **Cattle** | 750 | 18 (2.4) |
|  | **Sheep and goats** | 882 | 28 (3.2) |
|  | **Human** | 341 | 9 (2.6) |

**Table 2A:** Distribution of seroprevalence of *Brucella* antibodies among pastoral villages and cattle in the four studied districts of Borena zone (serial interpretation of RBT and C-ELISA)

| **District** | **Village** | **Species** | **N^o^ Sampled** | **N^o^ Positive (%)** |  |
| --- | --- | --- | --- | --- | --- |
| Gomole | Dhadacha Quufaa | Cattle | 26 | 1 (3.8) |  |
|  | Harboro | Cattle | 30 | 2 (6.7) |  |
|  | Dasie Gora | Cattle | 21 | 0 (0) |  |
|  | Bildim | Cattle | 32 | 0 (0) |  |
|  | Harobake | Cattle | 31 | 1 (3.2) |  |
| Elewoye | Elewoye Golba | Cattle | 32 | 2 (6.3) |  |
|  | Elewoye magala | Cattle | 40 | 2 (5.0) |  |
|  | Saba | Cattle | 44 | 5 (11.4) |  |
|  | Sarite | Cattle | 30 | 1 (3.3) |  |
|  | Ada Galchati | Cattle | 30 | 1 (3.3) |  |
| Dubuluk | Lafto | Cattle | 40 | 0 (0) |  |
|  | Higo | Cattle | 44 | 1 (2.3) |  |
|  | Arbale | Cattle | 24 | 1 (4.2) |  |
|  | Dhoqolle | Cattle | 50 | 0 (0) |  |
|  | Jigessa | Cattle | 55 | 1 (1.8) |  |
| Miyo | Baha | Cattle | 50 | 0 (0) |  |
|  | Arda Jila | Cattle | 41 | 0 (0) |  |
|  | Rarewardale | Cattle | 50 | 0 (0) |  |
|  | Boku | Cattle | 25 | 0 (0) |  |
|  | Chari Turura | Cattle | 54 | 0 (0) |  |
|  | Total | Cattle | 750 | 18 (2.4) | |

**Table 2B:** Distribution of seroprevalence of *Brucella* antibodies among pastoral villages and sheep and goats in the four studied districts of Borena zone (serial interpretation of RBT and C-ELISA)

| **District** | **Village** | **Species** | **N^o^ Sampled** | **N^o^ Positive (%)** |
| --- | --- | --- | --- | --- |
| Gomole | Dhadacha Quufaa | Sheep and goats | 31 | 0 (0) |
|  | Harboro | Sheep and goats | 32 | 0 (0) |
|  | Dasie Gora | Sheep and goats | 34 | 0 (0) |
|  | Bildim | Sheep and goats | 32 | 0 (0) |
|  | Harobake | Sheep and goats | 37 | 2 (5.4) |
| Elewoya | Elewoye Golba | Sheep and goats | 34 | 0 (0) |
|  | Elewoye magala | Sheep and goats | 48 | 3 (6.3) |
|  | Saba | Sheep and goats | 43 | 10 (23.3) |
|  | Sarite | Sheep and goats | 50 | 0 (0) |
|  | Ada Galchati | Sheep and goats | 40 | 0 (0) |
| Dubuluk | Lafto | Sheep and goats | 45 | 0 (0) |
|  | Higo | Sheep and goats | 35 | 0 (0) |
|  | Arbale | Sheep and goats | 58 | 2 (3.4) |
|  | Dhoqolle | Sheep and goats | 65 | 1 (1.5) |
|  | Jigessa | Sheep and goats | 47 | 0 (0) |
| Miyo | Baha | Sheep and goats | 50 | 2 (4.0) |
|  | Arda Jila | Sheep and goats | 50 | 1 (2.0) |
|  | Rarewardale | Sheep and goats | 50 | 6 (12.0) |
|  | Boku | Sheep and goats | 50 | 0 (0) |
|  | Chari Turura | Sheep and goats | 50 | 1 (2.0) |
|  | Total | Sheep and goats | 882 | 28 (3.2) |
