# Supplementary material for: Brucellosis in ruminants and pastoralists in Borena, Southern Ethiopia
Source: PLoS Negl Trop Dis. 2020 Jul 24;14(7):e0008461. doi: 10.1371/journal.pntd.0008461 (PMC7406081; doi:10.1371/journal.pntd.0008461)
Supplement: S1 Text — Approval from Addis Ababa University, Aklilu Lemma Institute of Pathobiology, Institutional Review Board. (PDF) [file pntd.0008461.s002.pdf]

Addis Ababa University
Aklilu Lemma Institute of Pathobiology
Institutional Review Board

Protocol amendment Approval Sheet

Minute No. ALIPB/IRB/004/2017/18
Date: 05 Thursday January, 2018

Title of the Project: **'Epidemiology of Brucellosis and Its Public Health Significance in Urban and Periurban Dairy Production System in Addis Ababa and selected districts of Borena Pastoral zone, Ethiopia'**

PI: Bedaso Mammo

Recommendation of the ALIPB Institutional Review Board

The PhD proposal was approved by ALIPB IRB (Minutes Ref No.: ALIPB/IRB/011/2015/16, Date 8th November 2016). The student requested protocol amendment pertaining to study sites with plausible justification. The ALIPB IRB discussed the importance of the requested protocol amendment. The Institutional Review Board reminds the student to submit progress reports of the work every 6 months and the final report upon completion of the study. Furthermore, the student should also notify the ALIPB/IRB ahead of time any amendments or modifications in the protocol or premature suspension or termination of the study

STATUS: APPROVED

IRB Chairperson: Dr. Tilahun Teklehaymanot
Signature: [illegible] 15/01/2018

IRB Secretary: Dr Lemu Golassa
Signature: [illegible] 05/01/18

Approval

Name: Dr Mirutse Giday, Director
Signature: [illegible]
Date: 15/01/18

CC/
Bedaso Mammo , ALIPB
Director's Office, ALIPB
ALIPB/IRB office
